# Correlations Between Sensory Evaluations and Instrumental Measurements in Milk Chocolate with Varying Emulsifier Levels and Particle Sizes

**DOI:** 10.3390/foods15050938

**Published:** 2026-03-07

**Authors:** Burcu Sasmaz, Gurbuz Gunes

**Affiliations:** Department of Food Engineering, Faculty of Chemical and Metallurgical Engineering, Istanbul Technical University, 34469 Maslak, Istanbul, Türkiye; akbasburc@itu.edu.tr

**Keywords:** milk chocolate, rheology, hardness, tribology, friction factor, saliva, Stribeck curve, sensory assessment, quantitative descriptive analysis, consumer acceptance test

## Abstract

This study was conducted to investigate and identify correlations among sensory and comprehensive consumer test results with rheological, textural, and tribological properties of milk chocolate in response to varying levels of particle size and emulsifier. To simulate realistic oral conditions, artificial saliva was incorporated into instrumental analyses. Rheological analysis revealed that increasing particle size and emulsifier concentration significantly reduced plastic viscosity, while emulsifier concentration alone increased yield stress due to structural reorganization within the fat phase. Tribological measurements demonstrated that larger particles increased friction in boundary and mixed lubrication regimes, whereas emulsifiers reduced friction in these regimes by enhancing fluid film formation. Under elastohydrodynamic conditions and with artificial saliva, friction was more influenced by the interaction between particle size and emulsifier level. Textural analysis showed that both parameters significantly influenced hardness, with saliva further softening the samples, especially those with higher emulsifier levels. Sensory evaluations indicated that emulsifiers enhanced flavor release and mouthfeel attributes, while smaller particles contributed to smoother texture and more balanced flavor perception. Consumer acceptance tests confirmed that samples with smaller particles and higher emulsifier levels received the highest scores in overall liking, taste, and texture. Instrumental parameters strongly correlated with key sensory attributes, with yield stress showing the highest positive associations with creaminess, smoothness, fat/milk flavor, and liking, while higher viscosity and friction were negatively linked to flavor release and mouthfeel. Instrumental hardness negatively correlated with cacao intensity and astringency, while saliva-induced softening was positively associated with sweetness and liking, highlighting the role of dynamic oral softening.

## 1. Introduction

Chocolate is composed of solid particles suspended in a continuous fat matrix, typically cocoa butter or its equivalents. Depending on the type—dark, milk, or white chocolate—it contains varying proportions of cocoa solids, sugar, and milk powder along with emulsifiers [1]. One of chocolate’s unique characteristics is its phase transition: it remains solid at room temperature but transforms into a viscous fluid at oral temperature due to the melting of its fat component, which binds the dispersed particles [2,3].

Understanding the flow behavior of chocolate is essential for achieving high-quality products with desirable texture. Rheological properties significantly influence viscosity, consistency, and mouthfeel, and they also affect aroma perception during oral processing [4]. Plastic viscosity, in particular, plays a critical role in processing operations such as pumping, filling, coating, and molding, as well as sensory attributes [5,6]. Moreover, particle size distribution, fat crystal structure, and ingredient composition all have a significant impact on chocolate texture, which in turn affects its rheological behavior, mouthfeel, and consumer perception [7]. However, rheological and texture profile analyses alone may not fully capture the sensory attributes of semi-solid foods like chocolate. Features such as creaminess, smoothness, and mouth coating are better explained through a combination of rheological and tribological approaches [8,9]. During oral processing, as the chocolate film between the tongue and palate is sheared and thinned, tribological mechanisms become dominant [10].

Tribology, the study of friction, wear, and lubrication between interacting surfaces in relative motion, is highly relevant to oral processing. In the mouth, interactions such as tongue-palate and tongue-food play a crucial role in texture perception [11]. Although tribological studies on biopolymers are well established, systematic tribological characterization of chocolate remains limited. In a study stimulating a tongue–palate contact to measure the friction coefficient showed that increasing cocoa solids content led to a maximum initial friction coefficient [9]. A new test method, simulated tongue–palate interactions, was investigated to show that the friction coefficient of molten chocolate increases with higher cocoa solids content and decreases with high levels of micro-areation [10]. Furthermore, a rapid tribological method was investigated to evaluate chocolate smoothness. Five chocolates with different cocoa concentrations were evaluated in terms of viscosity and using the average coefficient of friction via the addition of artificial saliva as a reliable proxy for oral conditions [12]. Another study reveals that increasing micro-aeration in chocolate significantly lowers the coefficient of friction in a stimulated oral environment, which directly relates to the sensory perception of a less sticky product [13]. The role of fat content is also significant in lubrication transitions. In mixed and elastohydrodynamic lubrication, transitions are governed by shear-thinning linked to aggregate breakdown and by selective particle entrainment or exclusion as a function of gap height, effects that become more pronounced in chocolate with higher solid content due to particle aggregation [14].

Oral processing of chocolate involves complex stages, including melting, mixing with saliva, and bolus formation. Saliva softens particles, lubricates oral surfaces, and facilitates bolus cohesion. These transformations shift the material behavior from solid-like to fluid-like, where tribological and rheological properties become dominated [10,12,15]. Saliva also influences taste and texture perception by reducing friction and interacting with polyphenols, contributing to astringency through increased friction mechanisms [16,17].

Sensory studies, including Quantitative Descriptive Analysis (QDA) or Temporal Dominance Sensations (TDSs), have linked food structure to consumer perception [13,18,19]. However, sensory evaluation alone does not sufficiently capture the complex mechanical and tribological changes that occur during the oral processing of chocolate. To achieve a comprehensive understanding of food behavior in the mouth, it is essential to integrate sensory analysis with instrumental rheological, textural, and tribological measurements [13,20,21].

Particle size and emulsifier level significantly affect the rheological and sensory properties of milk chocolate. Smaller particles increase surface area and viscosity, while a bimodal particle size distribution improves packing efficiency and reduces viscosity [7]. Emulsifiers like lecithin, polyglycerol polyricinoleate (PGPR), and ammonium phosphatide (AMP) enhance flow by reducing friction between particles; PGPR and AMP are especially effective at lowering yield stress even at low concentrations [22,23].

Although previous studies have examined individual effects of specific ingredients or processing variables on chocolate rheology, tribology, or texture, there is a lack of studies involving a comprehensive approach that integrates rheological, textural, tribological, and sensory analyses while simulating the actual oral processing conditions, particularly with the inclusion of saliva. Existing tribology studies on chocolate are limited in number and rarely relate frictional behavior to sensory perception, and rarely incorporate saliva into textural and tribological measurements. Moreover, there is a lack of information on correlations of comprehensive sensory and consumer test results with saliva-included instrumental measurements, such as rheology, texture, and tribology, in chocolate research. Identification of such correlations would be useful in further elaboration of mechanisms involved in sensory and consumer tests, as well as in developing models to predict sensory perceptions by instrumental analysis.

Thus, the aim of the present study was to investigate and identify correlations between comprehensive sensory evaluations and the rheological, textural, tribological properties of milk chocolate assessed with and without saliva in response to varying levels of particle size and emulsifier.

## 2. Materials and Methods

### 2.1. Materials

The ingredients used for chocolate preparation were supplied by Ulker Biskuvi Topkapı Chocolate Factory (Istanbul, Turkiye). Artificial saliva SAE0149 (Sigma Aldrich, St. Louis, MO, USA), composed of sodium chloride, potassium phosphate monobasic, potassium chloride, potassium thiocyanate, urea and adjusted to pH ~6.8, was utilized in instrumental analysis, including textural and tribological analysis. Polydimethylsiloxane (PDMS) sheets (Simpore, West Henrietta, NY, USA) were used in tribology analysis.

### 2.2. Methods

#### 2.2.1. Production of Chocolate Samples

Samples of milk chocolate varied with three distinct particle sizes and two different emulsifier levels were produced in a laboratory using a completely randomized experiment design. The highest and lowest emulsifier levels were chosen to represent the milk chocolate’s greatest variation in texture and flavor [24]. The ingredients were sucrose, cocoa butter, skimmed milk powder, cocoa liquor, whey powder, anhydrous milk fat, vegetable fats (palm and shea), polyglycerol polyricinoleate (PGPR) (0.05%), and flavoring agents. Initially, the components were thoroughly blended in a high-shear mixer (Inoksan 25M, Bursa, Turkiye) to achieve a homogeneous mass. Subsequently, the mixture underwent multi-stage refining using Bühler SDY300 (Bühler AG, Uzwil, Switzerland), resulting in the final chocolate formulations with three distinct particle size distributions with D_90_ particle sizes of 20 µm, 35 µm, and 50 µm (Table S1 and Figure S1). The particle size distribution of the final chocolate samples was determined by laser diffraction using a Helos^®^ BR laser diffraction particle size analyzer equipped with a Quixel wet dispersion unit to verify the targeted particle size (Sympatec GmbH, Clausthal-Zellerfeld, Germany) [25]. These refined mixtures were then flaked for further experimental trials. The flaked mixture was conched using Bühler Frisse ELK 0005-V (Bühler AG, Uzwil, Switzerland) for 150 min at a constant temperature of 60 °C. During the last 30 min of the conching process, ammonium phosphatide (AMP) and additional cocoa butter were incorporated. Specifically, 0.2% and 0.8% (*w*/*w*) AMP were added to the three distinct particle-sized samples, respectively. Following conching, tempering was performed using a Selmi Color EX tempering machine (Selmi S.r.l., Pollenzo, Italy) to ensure proper crystallization and gloss. The tempering index was carefully maintained between 4.0 and 6.0 to ensure optimal crystallization. For the molding step, the chocolate dosage was manually applied into rectangular molds (90 g) in the laboratory. Finally, the molded samples were packaged manually in 12-micron aluminum foil wrappers and stored at 19–20 °C until analysis. The production of the samples was replicated twice, as shown in Table 1. Detailed composition of milk chocolate samples was given in Table S2.

#### 2.2.2. Instrumental Measurements

##### Rheological Measurements

Rheological properties of the samples were measured using a rheometer (Anton Paar MCR302e, Graz, Austria). A cylindrical probe was used to measure the rheological properties, with system temperature controlled using a Peltier unit and a water bath. Temperature was maintained at 40 °C throughout the test using a Peltier system supported by a circulating water bath to ensure thermal stability. Chocolate samples were kept in an oven at 50 °C for 75 min to melt all the fat they contain. Samples were pre-sheared at 5 s^−1^ at 40 °C for 2 min before starting the measurement cycle. Shear stress was measured as a function of increasing shear rate from 5 s^−1^ to 60 s^−1^ for 2 min. Three replicates were carried out for the tests. The data were fitted to the Casson model to obtain yield stress and plastic viscosity:

(1) $$\sqrt{\tau }=\sqrt{\tau_{0}}+\sqrt{\mu *}\dot{\gamma },$$

where the following is true:

τ = shear stress;

τ_0_ = yield stress (minimum stress needed to initiate flow);

μ = plastic viscosity (resistance to flow once it starts);

$\dot{\gamma }$ = shear rate (rate of deformation).

##### Tribological Measurements

**Figure 1:**
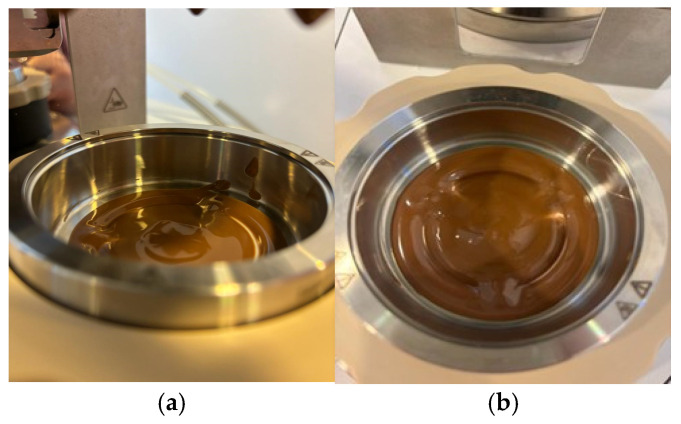
Melted chocolate samples during (**a**) and after test (**b**) without saliva in tribology measurement.

**Figure 2:**
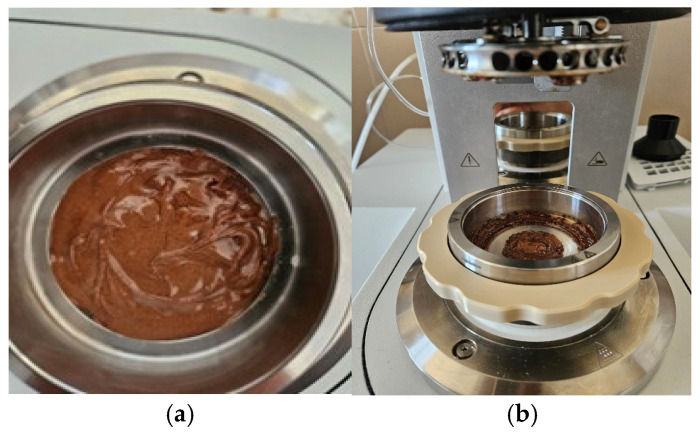
Melted chocolate samples during (**a**) and after test (**b**) with saliva in tribology measurement.

Friction force was measured during reciprocating motion of 3 balls on a plate using the Anton Paar MCR 302e rheometer with a tribo apparatus (Anton Paar GmbH, Graz, Austria). PDMS surface and steel balls were used in the tribo apparatus for the measurement [26,27]. A total of 7.5 g of melted chocolate was placed in the tribo apparatus, and the measurements were conducted at 37 °C to mimic oral conditions. The normal load was selected as 1 N during the measurements upon preliminary testing. The sliding velocity was varied from 10^−8^ to 1 m s^−1^ to see the frictional behavior of the tested samples in a wide range of velocities [28]. For assessing the impact of saliva, 1 mL of artificial saliva pre-heated to 37 °C was added to the same amount of sample (7.5 g melted chocolate) immediately before the measurement and manually mixed for 10 s using a stainless-steel stirring rod. The appearance of the melted chocolate samples during and after the test is presented in Figure 1 and Figure 2. A Stribeck curve (friction factor vs sliding velocity) was obtained in each test, and the curves were divided into 3 distinct regimes (Boundary, Mixed and Hydrodynamic) regarding the lubrication behavior of the samples [28]. The average friction coefficients were evaluated based on these 3 distinct regimes. The measurement was repeated three times for each chocolate sample.

##### Textural Measurements

**Figure 3:**
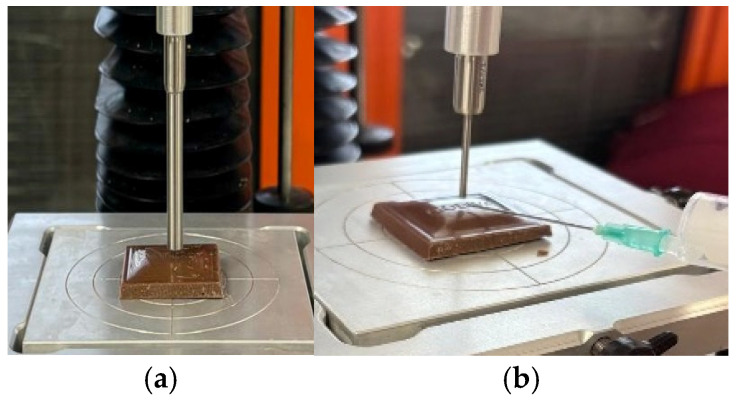
Hardness measurement of chocolate samples without saliva (**a**) and with saliva (**b**).

The penetration test was conducted to obtain the hardness of the chocolate by using texture analyzer (TA.XTplus, Stable Micro Systems Ltd., (Surrey, UK). Chocolate samples with 25 × 35 × 7 mm thickness were penetrated by a 2 mm diameter flat stainless-steel probe using the machine with a load cell of 50 N. The probe punctured each sample to a depth of 3.5 mm at a speed of 1 mm/s at ambient temperature. The trigger force was set at 5 g. This test procedure was also applied to samples with saliva: a 0.5 mL heated (37 °C) artificial saliva was injected onto the chocolate surface immediately before the measurements (Figure 3). The maximum force (N) recorded during the test was obtained using the TA Exponent software Version 6.2 integrated with the texture analyzer and designated as the hardness. The measurement was repeated three times for each chocolate sample.

#### 2.2.3. Sensorial Measurements

##### Quantitative Descriptive Analysis (QDA)

Quantitative Descriptive Analysis (QDA) was performed in the frame of sensorial analysis using 10 trained panelists. The chocolate samples were evaluated under four groups of parameters: taste (flavor), texture (mouthfeel), after-taste, and after-effect, as described in Table 2. The panelists were trained for the attributes and their definitions to ensure consistent scaling of the sample attributes. Each sample was placed in a plastic cup labelled with a three-digit random numbers. White light was used, and the presentation order of the samples was randomized in each session. In sensory booths, each sample was distributed to the panelists three times using distinct three-digit numbers. The panelists evaluated each sample’s attributes using a 12 cm line scale on a computer screen. The data recorded were analyzed using Redjade (RedJade Sensory Software Solutions LLC, USA) sensory software.

##### Consumer Acceptance Test

A total of 396 chocolate consumers participated in the study. Recruitment criteria required participants to be between 18 and 50 years of age and to represent an equal gender distribution (50% female, 50% male). Based on their self-reported consumption habits, participants were categorized prior to testing as heavy users (consuming chocolate at least once per day; 50% of the panel) and medium users (consuming chocolate at least once per week; 50% of the panel). The participants scored three attributes of the samples, namely ‘overall liking (considering all aspects such as appearance, aroma, taste and texture)’, ‘overall liking of the taste’, and ‘overall liking of the texture (considering firmness/softness and melting in the mouth)’, on a 9-point hedonic scale (9: like extremely; 1: dislike extremely) in the test [29]. A balanced incomplete block design was employed in the test in which each chocolate sample was evaluated by 66 consumers (block), and each pair of samples was evaluated within each block 6 times [30,31]. The participants were instructed to cleanse their palates with warm water and an unflavored cracker between each sample.

### 2.3. Statistical Evaluations

The effects of particle size and emulsifier type on the quality attributes of the milk chocolate samples were statistically evaluated with General Linear Model and one-way ANOVA using software (Minitab 22). Since consumer data were collected using a Balanced Incomplete Block Design (BIBD), the data were analyzed using a mixed-effects ANOVA model, with emulsifier and particle size as fixed factors and consumer as a random factor. Post hoc pairwise comparisons among the samples were performed using Tukey’s test. Statistical significance was determined at levels of *p* < 0.05 and *p* < 0.01. Pearson correlation analysis was conducted to explore the relationships between sensory data (QDA and consumer test results) and instrumental measurements (texture, rheology, and tribology). Rheological data were subjected to regression analysis to determine the parameters of the Casson model.

## 3. Results and Discussions

### 3.1. Rheological Measurements

Plastic viscosities (PV) and yield stress (YS) values are shown in Table 3. The Casson model fit the data with high goodness-of-fit across all samples (R^2^ > 0.98). Both PV and YS decreased markedly as the particle size increased from 20 to 50 micrometers (*p* < 0.05). This is thought to be associated with the reduction in the surface area, which must be coated with the continuous phase of fat. The reduced specific surface area requires less fat for particle lubrication and increases free fat in the product, resulting in lower PV. Moreover, larger particle size increases the interparticle distance, reduce particle–particle contacts, which makes it easier to break the structure and initiate flow, causing a reduction in YS [6,32].

However, as the concentration of emulsifier increased, PV decreased significantly because lower interparticle friction causes chocolate to flow with lower resistance. On the other hand, YS increased dramatically as the amount of emulsifier increased. This may be due to emulsifier–emulsifier interactions in the continuous phase, which might rearrange the sample’s microstructure [33].

In addition, the findings indicated that there was a significant interaction between the particle size and emulsifier level on rheological properties of milk chocolate (*p* < 0.05). It was found that increasing the particle size reduced the PV more effectively at a lower emulsifier level than at a higher one. Emulsifiers help the hydrophilic particle surface interact with the hydrophobic fat phase, preventing phase separation. However, because mechanical resistance is already significant in the presence of big particles, the emulsifier’s influence becomes secondary. Furthermore, the reduced surface area of larger particles limits the adsorption capacity of emulsifiers [34]. Compared with systems with smaller particles, emulsifiers may be far less effective at reducing the YS of chocolate with large particles [35].

### 3.2. Tribological Measurements

**Figure 4:**
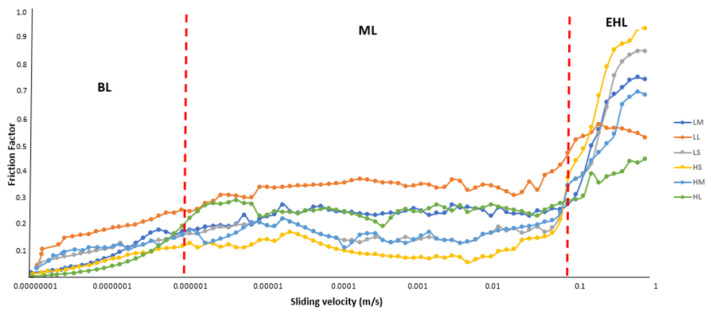
The Stribeck curves of samples without saliva affected by emulsifier and particle size.

The Stribeck curves for the chocolate samples without saliva are shown in Figure 4. Stribeck curves are divided into 3 regimes, namely boundary lubrication (BL), mixed lubrication (ML), and elastohydrodynamic lubrication (EHL), in terms of the samples’ lubrication behavior [28]. As illustrated in Table 4, the average friction factors were computed using three distinct regimes. An increase in particle size resulted in a significant increase in the average friction factor in BL and ML regions (*p* < 0.05). This is consistent with a previous study, which suggests that oversized surface features caused increased friction in BL and ML regimes by promoting lubricant film collapse and reducing oil entrainment after reversal [36]. Conversely, increasing particle size resulted in a significant reduction in the average friction factor in the EHL region (*p* < 0.05). When the lubricant film thickness becomes comparable to particle size, larger particles are mostly kept out of the contact zone, allowing the fat phase to dominate the sliding interface. This behavior aligns with classical tribological descriptions of regime evolution in which increasing entrainment speed and film thickness progressively decouple surface roughness from the interface, thereby lowering friction upon entry into EHL [37]. On the other hand, an increase in emulsifier level caused a reduction in the average friction factor in BL, ML, and EHL regions (*p* < 0.05). The presence of emulsifiers, particularly at higher concentrations, mitigated this effect in ML by reducing solid–solid interactions and enhancing fluid film stability [38]. No interaction between the emulsifier and particle size was observed in the BL and ML regions (*p* > 0.05), showing their independent impacts on the average friction factors in these regions. However, the average friction factor in the EHL region decreased more sharply with increasing particle size at high emulsifier levels than at low emulsifier levels (*p* < 0.10). Previous mixed-EHL modeling studies have demonstrated that solid particle size and concentration can significantly alter load sharing, film thickness, and frictional response under elastohydrodynamic conditions [39]. Moreover, the friction behavior in high-shear EHL regimes is strongly governed by the effective viscosity and pressure–viscosity response of the lubricant, which may enable the formation of low-shear layers within the contact [40].

All in all, the sample with the higher emulsifier level and the smaller particles showed the lowest coefficient of friction within the slow (BL regime) and intermediate sliding velocity (ML regime). However, in the EHL regime, i.e., fast sliding velocity, the sample with a high emulsifier level and small particle showed the highest friction factor.

**Figure 5:**
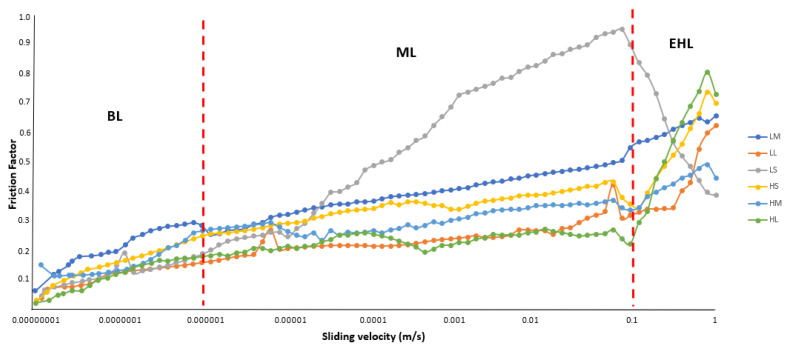
The Stribeck curves of samples with saliva affected by emulsifier and particle size.

The Stribeck curves for the samples with saliva were also obtained and are presented in Figure 5. From these data, the average friction factors of the samples with saliva were computed in the three distinct regimes (BL, ML, EHL) as reported in Table 5. In BL and EHL regimes, no systematic, meaningful decrease or increase was observed via changing the emulsifier level and particle size (*p* > 0.05). In the ML regime, increasing the emulsifier level and increasing particle size resulted in a reduction of the average friction factor with saliva (*p* < 0.05). On the other hand, there was a significant interaction between emulsifier level and particle size on average friction factor under saliva in ML (*p* < 0.05). At a lower emulsifier level, an increase in particle size was found to be more effective in the reduction of the average friction factor of the samples with saliva in the ML regime compared with the higher emulsifier level. The presence of saliva appeared to buffer the effects of particle size and emulsifier level, reducing the significance of individual factors in BL and EHL regimes. However, the interaction between these variables remained important, particularly in ML, where lower emulsifier levels combined with larger particles led to reduced friction factor. This may be due to saliva’s role in modifying surface interactions and enhancing lubrication through mucin-like components [41] In addition, these mechanisms are well documented in oral tribology studies on chocolate and chocolate–saliva boluses [14]. In such systems, saliva dilution leads to the formation of an oil-in-water bolus, where the aqueous phase or saliva-mediated fat redistribution can dominate friction behavior depending on particle size, viscosity ratios, and film thickness. Therefore, the reduced friction observed at low emulsifier levels may reflect enhanced saliva-mediated lubrication pathways, particularly when larger particles alter bolus structuring and promote more effective aqueous-phase film formation.

### 3.3. Textural Measurements

Hardness values of the samples with and without saliva are given in Table 6. Both the emulsifier level and the particle size significantly influenced the hardness of milk chocolate samples. As the emulsifier amount and particle size increased, the measured hardness values decreased (*p* < 0.05). This finding is consistent with previous research reporting that increasing lecithin concentration from 0.3% to 0.5% led to a reduction in the hardness of tempered dark chocolate [42]. Lecithin covers sugar and cocoa particles, diminishing interparticle adhesion and increasing void fraction within the matrix, which consequently reduces textural parameters such as hardness and cohesiveness [4,42]. An inverse relationship between particle size and chocolate hardness has also been reported in several studies, with coarser particle sizes leading to lower hardness values, as larger particles result in less efficient packing and a less compact solid network [43,44].

Importantly, the inclusion of artificial saliva in texture analysis introduced a notable shift in the hardness values of the samples. Samples analyzed under saliva exhibited lower hardness values compared with those tested without saliva, as illustrated by their difference in Table 6 (*p* < 0.05). Also, the difference in hardness between with and without saliva was found to be significantly higher in samples with a higher emulsifier level. Hydration by saliva can soften and swell solid-food particles, thereby altering their mechanical properties such as modulus and hardness. As bolus formation proceeds, the particles hydrate, soften, aggregate, and form the soft mass that is eventually swallowed [45]. This effect was more pronounced in formulations with higher emulsifier content in our study. Emulsifiers influence food–saliva interfacial interactions, which can indirectly affect lubrication and softening behavior during mastication [46]. Moreover, emulsifiers are known to modify the interfacial tension between dispersed particles and the continuous fat phase, which may facilitate breakdown and melting during mastication [10,47].

### 3.4. Sensorial Measurements

#### 3.4.1. QDA—Flavor Related Attributes

**Figure 6:**
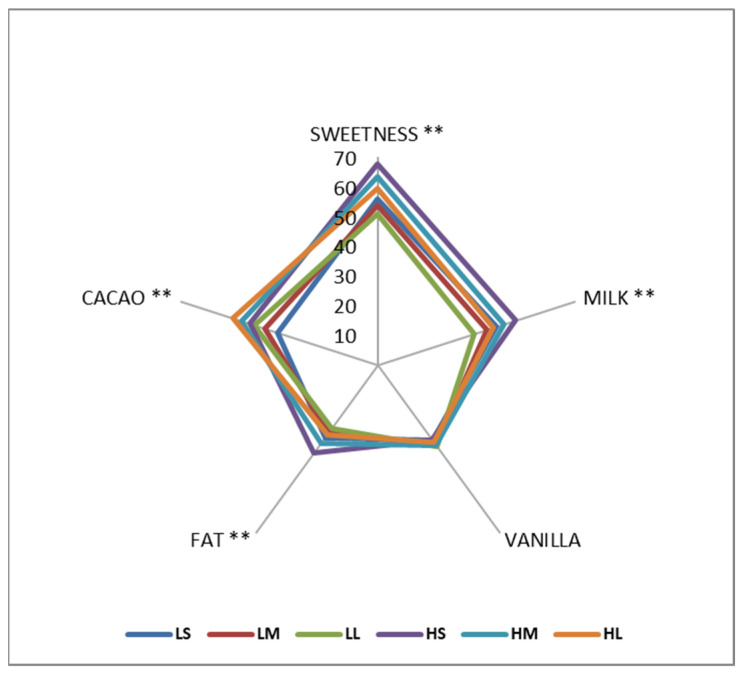
The spider map of flavor-related attributes for milk chocolate samples as affected by emulsifier level and particle size. (** indicates significant differences between the samples in 95% CL).

The QDA results for flavor-related attributes are given in Figure 6. An increase in emulsifier level significantly enhanced the perception of sweetness, milk, fat, and cocoa flavors (*p* < 0.05). This can be attributed to the emulsifier’s ability to improve the dispersion of fat and flavor compounds within the chocolate matrix, facilitating more efficient release and interaction with taste receptors during oral processing. Emulsifiers reduce interfacial tension and stabilize the fat phase, which enhances the solubilization and delivery of flavor-active compounds [48,49]. Another study also emphasized that emulsifiers contribute to improved melting behavior and flavor release, particularly in the milk chocolate system [23]. Consequently, the overall flavor intensity of milk chocolate increases with higher emulsifier levels.

No significant effect was observed with the interaction of emulsifier level and particle size on any of the flavor attributes (*p* > 0.05). This suggests that these two formulation variables influence flavor perception through distinct and independent mechanisms. While emulsifiers primarily affect the distribution and release of flavor compounds, particle size modulates the physical breakdown and dissolution behavior of chocolate during oral processing.

**Figure 7:**
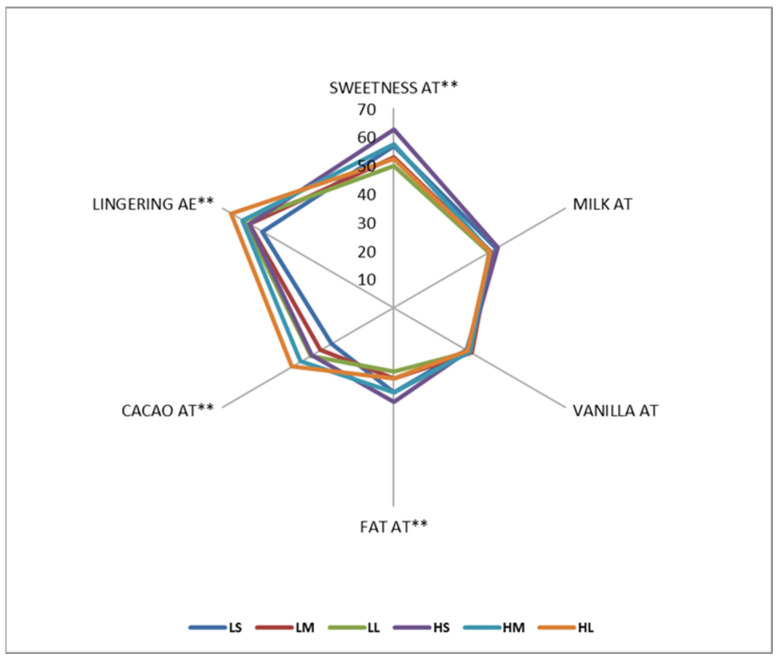
The spider map of aftertaste and aftereffect related attributes for milk chocolate samples as affected by emulsifier level and particle size (AT: After-taste and AE: After-effect, ** indicates significant differences between the samples in 95% CL).

The QDA result for after-taste (i.e., flavor remains in the mouth one minute after swallowing milk chocolate) and after-effect (i.e., sensation remains in the mouth one minute after swallowing milk chocolate) related attributes is given in Figure 7. Emulsifier concentration significantly increased the aftertaste intensity of sweetness, fat, and cocoa flavors (*p* < 0.05), but had no effect on milk aftertaste (*p* > 0.05). This indicates that the emulsifier’s impact on milk flavor perception diminishes over time, possibly due to rapid clearance or transformation of milk-related compounds in the oral cavity. Similarly, decreasing particle size significantly enhanced the after-taste of sweetness and fat flavors, while increasing particle size intensified cocoa aftertaste (*p* < 0.05). These findings demonstrate how particle dissolution dynamics shape residual taste sensations and are in line with the changes in flavor perception seen during eating. No significant effects were found for vanilla and milk aftertaste in relation to particle size (*p* > 0.05). Both emulsifier level and particle size were found to significantly influence the lingering sensation (*p* < 0.05). Higher emulsifier levels and larger particle sizes contributed to prolonged flavor retention in the oral cavity. This may be due to the enhanced adhesion of flavor compounds to oral surfaces by emulsifiers and slower solubilization rates of larger particles [46,48]. Smaller particles contributed to a smoother texture and more uniform flavor release, enhancing creaminess and sweetness perception.

#### 3.4.2. QDA—Texture Related Attributes

**Figure 8:**
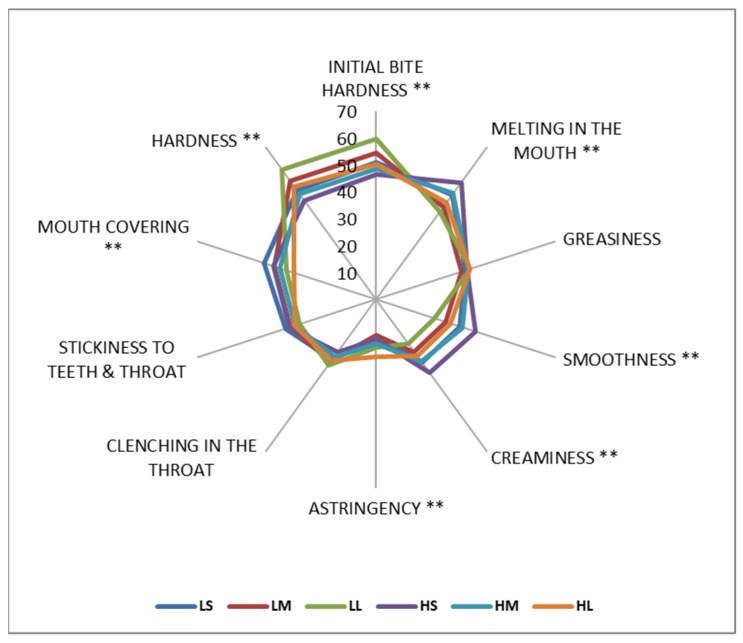
The spider map of texture-related attributes for milk chocolate samples as affected by emulsifier level and particle size. (** indicates significant differences between the samples in 95% CL).

The QDA results for texture-related attributes are given in Figure 8. Both the emulsifier level and particle size significantly influenced the sensorial textural attributes of milk chocolate. Increasing the emulsifier level led to a significant decrease in initial bite hardness, hardness, and mouth covering perceptions, while it caused an increase in melting in the mouth, smoothness, creaminess, and astringency perceptions (*p* < 0.05). Increasing emulsifier level can promote a softer structure and a more fatty, mouth-coating oral sensation by enhancing lubrication and fat phase mobility, particularly in well-tempered and non-bloomed chocolate systems [50]. As particle size increased, the initial bite hardness, hardness, and astringency increased, while melting in the mouth, smoothness, creaminess, and mouth covering attributes decreased (*p* < 0.05). These findings align with previous studies showing that larger particle sizes reduced the perceived smoothness and melting behavior of chocolate due to reduced surface area and altered rheological properties [51]. However, changes in emulsifier level and particle size did not show any effect in greasiness, stickiness to teeth and throat, and clenching in the throat (*p* > 0.05), suggesting that these attributes are less sensitive to formulation changes.

#### 3.4.3. Consumer Acceptance Test Results

Consumer acceptance test results are given in Table 7. As the emulsifier level increased, ‘overall liking’ (considering all aspects such as appearance, aroma, taste and texture), ‘overall liking of the taste’, and ‘overall liking of the texture’ (considering firmness/softness and melting in the mouth) of the samples increased significantly (*p* < 0.05). On the other hand, these three attributes decreased significantly with increasing particle size (*p* < 0.05). So, the sample with high emulsifier level and small particle size received the highest liking scores (over 7—liked very much or extremely), while the sample with low emulsifier level and large particle size had the lowest liking scores. However, none of the samples were scored at or below 5 (neither liked nor disliked); all of them were scored over 6 (liked slightly). Furthermore, the mixed-effects model ANOVA indicated that the random factor, consumer, was not significant, demonstrating that differences among the liking scores of the samples were driven primarily by the sample characteristics.

### 3.5. Correlation Between Instrumental and Sensory Parameters

**Figure 9:**
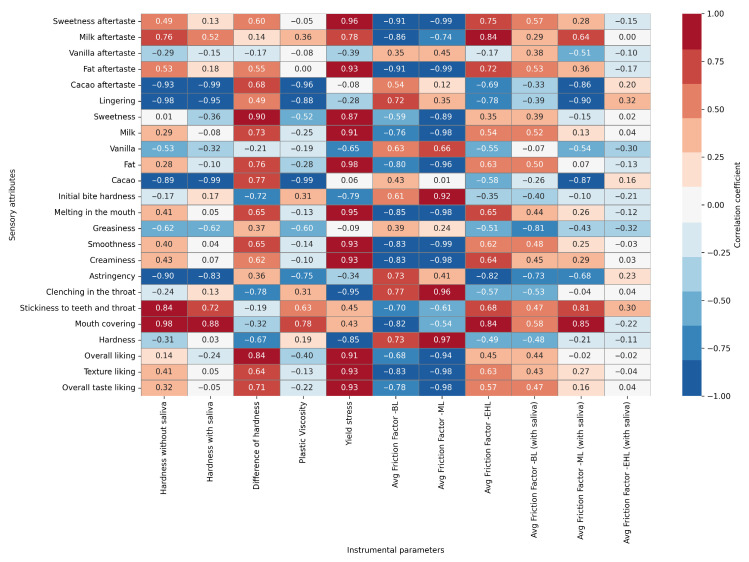
Heatmaps illustrating the correlation coefficients between the measured instrumental parameters and the sensory attributes of milk chocolate samples as affected by emulsifier level and particle size.

We also explored the correlations between sensory attributes and instrumental measurements (Figure 9). When the correlation between sensory flavor-related attributes and instrumental parameters was evaluated, one of the strongest positive correlations (r > 0.9) was observed between fat and milk flavor with yield stress values. These findings aligned with previous research highlighting the role of yield stress in delivering creamy and indulgent textures [52,53]. Also, yield stress exhibited the highest positive correlations (r > 0.9) with sensory descriptors such as sweetness aftertaste. Conversely, negative correlations (r > 0.9) were observed between plastic viscosity and instrumental hardness values with cocoa-related sensory attributes (cacao aftertaste and cacao flavor). This suggests that higher viscosity may hinder flavor release, consistent with the studies reporting that increased viscosity reduced volatile compound diffusion and flavor perception [54,55]. On the other hand, attributes such as milk flavor, vanilla flavor, fat flavor, and sweetness aftertaste were also inversely correlated (r > 0.9) with frictional parameters, reinforcing the hypothesis that lubrication plays a pivotal role in flavor release [11,55].

When the correlation between the sensory texture-related attributes and instrumental parameters was evaluated, one of the strongest positive correlations was observed between the mouth covering and the hardness, with and without saliva, supporting the findings that firmer structures promoted coating sensations and prolonged oral residence time [56]. Also, yield stress exhibited the highest positive correlations (r > 0.9) with sensory descriptors such as smoothness, creaminess and melting in the mouth. These results align with previous research indicating that higher yield stress contributed to enhanced creaminess perception by increasing structural integrity and controlled fat release during oral processing [6,7]. Furthermore, astringency was inversely correlated with yield stress and hardness values (r > 0.8), showing that structural firmness mitigates these undesirable sensations, a trend also noted in confectionery and dairy systems where higher structural stability reduces oral friction and irritation [57]. Tribological parameters provided additional insights into oral lubrication and texture perception. In particular, the average friction factor without saliva in BL and ML showed the highest correlations (r > 0.9) with the sensory attributes. Friction factor showed strong negative correlations with smoothness (r > 0.9), melting in the mouth (r > 0.9), and creaminess (r > 0.9), confirming that lower friction enhances perceived smoothness and creaminess. These results are consistent with recent tribology-based studies highlighting that frictional behavior during oral processing strongly affected sensory attributes such as silkiness and lubricity [58,59]. Interestingly, the presence of saliva reduced the strength of these correlations, suggesting that salivary lubrication partially masks differences in product tribology, as also noted in some dairy systems [60]. Furthermore, the average friction factor without saliva in ML had a strong positive correlation with hardness (r > 0.9) and mouth burning (clenching in the throat) (r > 0.9) sensations, suggesting that a higher friction factor contributes to undesirable mouthfeel sensations, such as dryness and roughness.

Consumer test measures such as texture liking and overall taste liking also demonstrated strong positive correlations with yield stress and difference of hardness (r > 0.8). Likewise, it was reported that increased friction caused negative texture perceptions like increased graininess and astringency. The review also highlights that yogurt samples exhibiting a higher coefficient of friction were sensorially described by consumers as “lumpy” and “chalky” [57]. Similar findings have been reported for chocolate and dairy systems, where moderate yield stress contributed to perceived creaminess and cohesiveness, while rapid breakdown under saliva enhanced overall mouthfeel perception [61]. Conversely, the strongest negative correlation was found between the consumer liking scores and average friction factor in BL and ML (r = 0.71–0.99), indicating that increased friction during oral processing negatively impacted consumer acceptance. This result is associated with elevated BL and ML friction with roughness, drag, and astringency. This increase in friction is attributed to the depletion of the lubricating film, which leads to direct surface contact and increased resistance during oral processing [62]. A systematic review also confirms that elevated friction coefficients in the boundary and mixed lubrication regimes are positively correlated with sensory attributes such as roughness, stickiness, and astringency [11].

Overall, the instrumental parameters—yield stress, plastic viscosity, hardness, and friction factors- showed strong correlations with key sensory attributes, highlighting their central role in shaping chocolate perception. Among these, yield stress emerged as the most influential instrumental parameter governing both flavor- and texture-related sensory perceptions, showing strong positive correlations with key descriptors, such as creaminess, smoothness, fat/milk flavor, and overall liking. In contrast, plastic viscosity and frictional parameters (particularly friction factor in BL and ML regimes) exhibited strong negative correlations with desirable sensory attributes and consumer acceptance, highlighting the detrimental effects of increased viscosity and oral friction on flavor release and mouthfeel. Tribological measurements further confirmed the pivotal contribution of lubrication dynamics, with high friction factors reducing smoothness, creaminess, and melting sensations. Hardness also showed important correlations: while absolute hardness (with and without saliva) correlated negatively with cacao intensity, lingering, and astringency, it correlated positively with mouth covering and stickiness to teeth in the absence of saliva. Conversely, the difference in hardness (hardness without saliva—hardness with saliva), representing saliva-driven softening, was positively associated with sweetness and overall liking, emphasizing that the dynamic structural transformation during oral processing—rather than static firmness alone—drives flavor delivery, mouthfeel, and consumer preference.

## 4. Conclusions

Particle size and emulsifier concentration significantly affected the rheological, textural, and frictional properties of milk chocolate. Sensorial evaluations and consumer tests revealed that higher emulsifier levels and smaller particles enhanced flavor release and mouthfeel attributes, leading to significantly higher scores of overall liking, taste, and texture.

The instrumental parameters—yield stress, plastic viscosity, hardness and friction factors- showed strong correlations with key sensory attributes strongly dictating chocolate perception. Yield stress emerged as the primary driver for flavor and texture, correlating positively with creaminess, smoothness, fat/milk flavor, and overall liking. In contrast, plastic viscosity and frictional factors (BL and ML regimes) negatively impacted mouthfeel, flavor release, and consumer acceptance. Tribological data confirmed that a high friction factor reduces smoothness, creaminess, and melting sensations. Notably, while static hardness showed mixed effects, the saliva-driven softening (hardness difference) was positively correlated with sweetness and overall liking. Ultimately, superior milk chocolate quality depends primarily on optimizing yield stress while minimizing frictional resistance.

Future work should focus on expanding sample sets and employing dynamic sensory analyses to map the mechanistic links between instrumental measurements and sensory evaluations. Such insights will enable the targeted design of products with superior mouthfeel by balancing texture, lubrication, and breakdown kinetics. Additionally, developing mathematical models to predict sensorial profiles from instrumental data would be highly valuable. Developing more advanced methods to incorporate the impact of saliva into instrumental characterization will also be crucial for accurately mimicking real oral processing and enhancing predictive power.

## Figures and Tables

**Table 1:** Technical details of milk chocolate samples and experimental design table.

Sample Code	Emulsifier Level (%)	D_90_ Particle Size (µm)	Replicate
LS	0.2	20	1
LS	0.2	20	2
LM	0.2	35	1
LM	0.2	35	2
LL	0.2	50	1
LL	0.2	50	2
HS	0.8	20	1
HS	0.8	20	2
HM	0.8	35	1
HM	0.8	35	2
HL	0.8	50	1
HL	0.8	50	2

**NOTE:** LS indicates low emulsifier and small particle. LM indicates low emulsifier and medium particle. LL indicates low emulsifier and large particle. HS indicates high emulsifier and small particle. HM indicates high emulsifier and medium particle. HL indicates high emulsifier and large particle.

**Table 2:** The attributes and the definitions used in the milk chocolates’ sensory evaluations with QDA.

Attributes	Definitions
Milk flavor (weak-strong)	The intensity of a milk taste, similar to packaged milk product, from weak to strong.
Cacao flavor (weak-strong)	The intensity of a cocoa taste, similar to baking cocoa powder, from weak to strong.
Vanilla flavor (weak-strong)	The degree of intensity of powder, like flower vanillin taste from less to more.
Fat flavor (weak-strong)	The degree of intensity of fat like butter taste from less to more.
Sweetness (weak-strong)	The degree of intensity of sweet taste from less to more.
Initial bite hardness (soft-hard)	The measure of the hardness of the initial bite, from slightly to very. The degree of slightly hard of apricot to very hard like hard candy.
Hardness (soft-hard)	Except the hardness of initial bite, the measure of the hardness during eating, from slightly to very. The degree of hardness from apricot to hard candy.
Melting in the mouth (slow-fast)	The measure of the melting speed of the chocolate in the mouth, from slow to fast.
Stickiness to teeth and throat (slightly-very)	The measure of how the chocolate is to stick to the teeth and gum during chewing, from slightly to very.
Clenching in the throat (slightly-very)	The measure of the physical sensation of the throat constricting due to too much sweetness, from slightly to very.
Astringency (slightly-very)	The measure of astringency, from slightly to very.
Mouth covering (slightly-very)	The intensity of covering the whole mouth from less to more.
Greasiness (slightly-very)	The degree of feeling felt in mouth as oily and slippery from less to more.
Smoothness (slightly-very)	The intensity of smooth feeling and texture of chocolate from slightly to very.
Creaminess (slightly-very)	The intensity of creamy feeling and texture of chocolate from slightly to very.
Milk after-taste (weak-strong)	The intensity of a milk taste, similar to packaged milk product, from weak to strong.
Cacao after-taste (weak-strong)	The intensity of a cocoa taste, similar to baking cocoa powder, from weak to strong.
Vanilla after-taste (weak-strong)	The degree of intensity of powder, like flower vanillin taste from less to more.
Fat after-taste (weak-strong)	The degree of intensity of fat like butter taste from less to more.
Sweetness after-taste (weak-strong)	The degree of intensity of sweet taste from less to more.
Lingering after-effect (short-long)	The measure of how long any remaining flavor lingers in the mouth, from short to long.

**Table 3:** Plastic viscosity (PV) and yield stress (YS) of the samples as affected by emulsifier and particle size.

Sample Code	Emulsifier Level (%)	D_90_ Particle Size (µm)	PV (Pa.s)	YS (Pa)
LS	0.2	20	2.27 ^a^	2.60 ^b^
LM	0.2	35	1.82 ^b^	1.84 ^c^
LL	0.2	50	1.47 ^c^	1.49 ^d^
HS	0.8	20	1.23 ^d^	4.81 ^a^
HM	0.8	35	1.06 ^e^	2.87 ^b^
HL	0.8	50	0.93 ^f^	1.88 ^c^

Values with different superscripts in the same column were significantly different at *p* < 0.05.

**Table 4:** Average friction factors of the samples as affected by emulsifier and particle size without saliva.

Sample Code	Emulsifier Level (%)	D_90_ Particle Size (µm)	Boundary Lubrication (BL)	Mixed Lubrication (ML)	Hydrodynamic Lubrication (EHL)
LS	0.2	20	0.14 ^bc^	0.18 ^c^	0.63 ^b^
LM	0.2	35	0.19 ^b^	0.24 ^b^	0.56 ^c^
LL	0.2	50	0.22 ^a^	0.31 ^a^	0.45 ^d^
HS	0.8	20	0.11 ^c^	0.11 ^d^	0.75 ^a^
HM	0.8	35	0.17 ^b^	0.16 ^c^	0.47 ^d^
HL	0.8	50	0.24 ^a^	0.24 ^b^	0.30 ^e^

Values with different superscripts in the same column were significantly different at *p* < 0.05.

**Table 5:** Average friction factors of the samples with saliva as affected by emulsifier and particle size.

Sample Code	Emulsifier Level (%)	D_90_ Particle Size (µm)	Boundary Lubrication (BL)	Mixed Lubrication (ML)	Hydrodynamic Lubrication (EHL)
LS	0.2	20	0.18 ^a^	0.82 ^a^	0.50 ^a^
LM	0.2	35	0.23 ^a^	0.35 ^b^	0.61 ^a^
LL	0.2	50	0.15 ^a^	0.25 ^c^	0.40 ^a^
HS	0.8	20	0.21 ^a^	0.34 ^b^	0.50 ^a^
HM	0.8	35	0.20 ^a^	0.25 ^c^	0.43 ^a^
HL	0.8	50	0.15 ^a^	0.18 ^d^	0.67 ^a^

Values with different superscripts in the same column were significantly different at *p* < 0.05.

**Table 6:** Hardness of the samples as affected by emulsifier and particle size.

Sample Code	Emulsifier Level (%)	D_90_ Particle Size (µm)	Hardness Without Saliva (gf)	Hardness with Saliva (gf)	Difference of Hardness (gf)
LS	0.2	20	2830.04 ^a^	2621.64 ^a^	208.4 ^f^
LM	0.2	35	2479.73 ^b^	2236.15 ^b^	243.6 ^e^
LL	0.2	50	2224.47 ^c^	1951.82 ^c^	272.6 ^d^
HS	0.8	20	2537.16 ^b^	1966.86 ^c^	570.3 ^a^
HM	0.8	35	2240.34 ^c^	1719.90 ^d^	523.0 ^b^
HL	0.8	50	2003.19 ^d^	1551.79 ^e^	451.4 ^c^

Values with different superscripts in the same column were significantly different at *p* < 0.05.

**Table 7:** Liking scores of the samples in the consumer test as affected by emulsifier and particle size.

Sample Code	Emulsifier Level (%)	D_90_ Particle Size (µm)	Overall Liking	Overall Taste Liking	Overall Texture Liking
LS	0.2	20	7.23 ^c^	7.12 ^b^	7.32 ^ab^
LM	0.2	35	6.97 ^cd^	6.82 ^bc^	6.87 ^cd^
LL	0.2	50	6.71 ^d^	6.53 ^c^	6.66 ^d^
HS	0.8	20	7.94 ^a^	7.58 ^a^	7.71 ^a^
HM	0.8	35	7.64 ^ab^	7.21 ^ab^	7.34 ^ab^
HL	0.8	50	7.28 ^bc^	7.02 ^b^	7.06 ^bc^

Values with different superscripts in the same column were significantly different at *p* < 0.05. (9-point scale: 1: dislike extremely, 5: neither liked or disliked, 9: liked extremely).

## Data Availability

The original contributions presented in the study are included in the article/Supplementary materials. Further inquiries can be directed to the corresponding author.

## Supplementary Materials

The following supporting information can be downloaded at: https://www.mdpi.com/article/10.3390/foods15050938/s1, Table S1. The PSD results of the milk chocolate samples. Figure S1. The examples of PSD graphs for **a**. LS, **b**. HS, **c**. LM, **d**. HM, **e**. LL, **f**. HL. Table S2. Composition of milk chocolate samples (based on 100 grams).
