# Supplementary material for: Correlations Between Sensory Evaluations and Instrumental Measurements in Milk Chocolate with Varying Emulsifier Levels and Particle Sizes
Source: Foods. 2026 Mar 7;15(5):938. doi: 10.3390/foods15050938 (PMC12984600; doi:10.3390/foods15050938)
Supplement: Supplementary file 1 [file foods-15-00938-s001.zip › foods-4114983-supplementary.pdf]

Supplemented Materials

1. Particle Size Distribution (PSD) of Chocolates

1.1. Method for PSD of Milk Chocolate Samples

The particle size distribution of samples was measured by laser diffraction using a Helos® BR laser diffraction particle size analyzer with a Quixel wet dispersion unit (Sympatec GmbH, Clausthal-Zellerfeld, Germany).

About 0.2 g of milk chocolate was dispersed in sunflower oil. Sunflower oil was filled into the chamber as a carrier medium, and the internal temperature was adjusted to 40 °C. The mixer speed was set to 30 rpm. The sample was subjected to ultrasonic treatment for 2 min to achieve complete particle separation, after which dispersion was maintained by continuous stirring throughout the measurement. Particle size distribution was expressed as the relative volume of particles with different sizes and presented as distribution curves (Paqxos Software for Helos BR Qiuxel). Particle size distribution (PSD) analysis provided information on volume fractions of particles less than specified sizes: D_{90}, 90% of the volume consists of particles smaller than this size; D_{50}, 50% of the volume consists of particles with this size; D_{10}, 10% of the volume consists of particles smaller than this size. All the measurements were done with three replicates.

1.2. Results for PSD of Milk Chocolate Samples

The PSD analysis was also performed to samples, as shown in Table S1. It can be understood that the particle size was achieved, which was aimed at the experimental design. When d(0.1) and d(0.9) values were evaluated together, it was observed that the widest particle size distribution belonged to HL and LL samples. The example of particle size distribution graphs was given in Figure S1 belong to representative samples for each batch.

Table S1. The PSD results of the milk chocolate samples

**Sample Code**	D_{10}	D_{50}	D_{90}
LS	1.8^{b}	7.4^{c}	23.5^{c}
LS	1.9^{b}	7.3^{c}	22.5^{c}
LM	2.2^{b}	8.7^{b}	33.6^{b}
LM	2.1^{b}	8.6^{b}	34.5^{b}
LL	4.3^{a}	12.4^{a}	50.8^{a}
LL	4.2^{a}	12.1^{a}	50.4^{a}
HS	1.6^{b}	7.1^{c}	21.7^{c}
HS	1.5^{b}	6.9^{c}	21.3^{c}
HM	2.4^{b}	9.4^{b}	36.7^{b}
HM	2^{b}	9.1^{b}	35.4^{b}
HL	3.9^{a}	11.7^{a}	48.5^{a}
HL	3.5^{a}	11.2^{a}	49.4^{a}

***NOTE:** ***LS** indicates low emulsifier and small particle. **LM** indicates low emulsifier and medium particle. **LL** indicates low emulsifier and large particle. **HS** indicates high emulsifier and small particle. **HM** indicates high emulsifier and medium particle. **HL** indicates high emulsifier and large particle.*

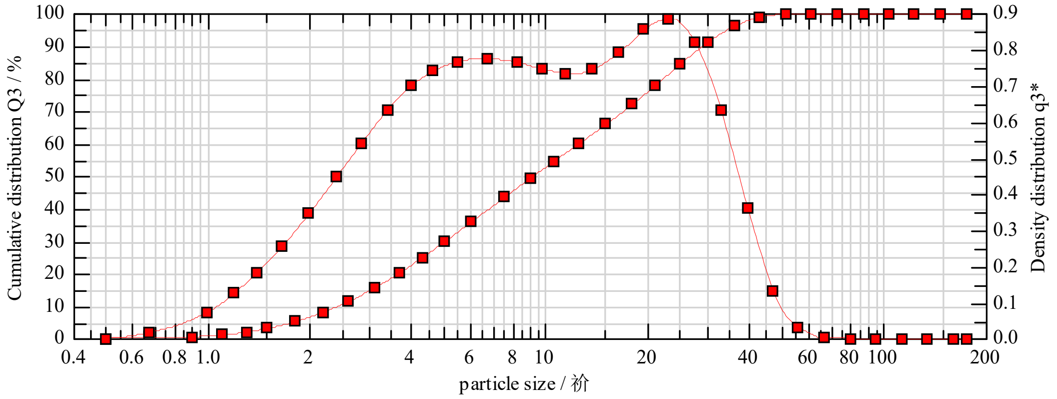
Cumulative distribution Q3 / %
Density distribution q3*
particle size / 祄
0 10 20 30 40 50 60 70 80 90 100
0.0 0.1 0.2 0.3 0.4 0.5 0.6 0.7 0.8 0.9
0.4 0.6 0.8 1.0 2 4 6 8 10 20 40 60 80 100 200

(a)

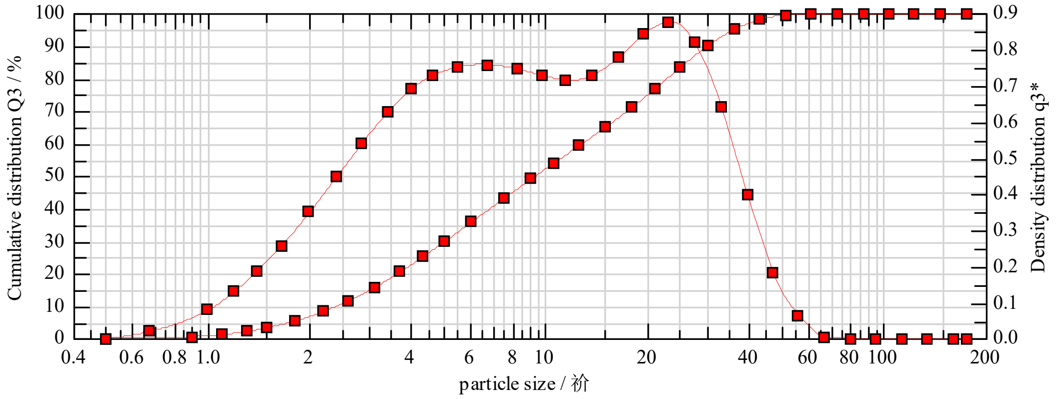
Cumulative distribution Q3 / %
Density distribution q3*
particle size / 祄
0 10 20 30 40 50 60 70 80 90 100
0.0 0.1 0.2 0.3 0.4 0.5 0.6 0.7 0.8 0.9
0.4 0.6 0.8 1.0 2 4 6 8 10 20 40 60 80 100 200

(b)

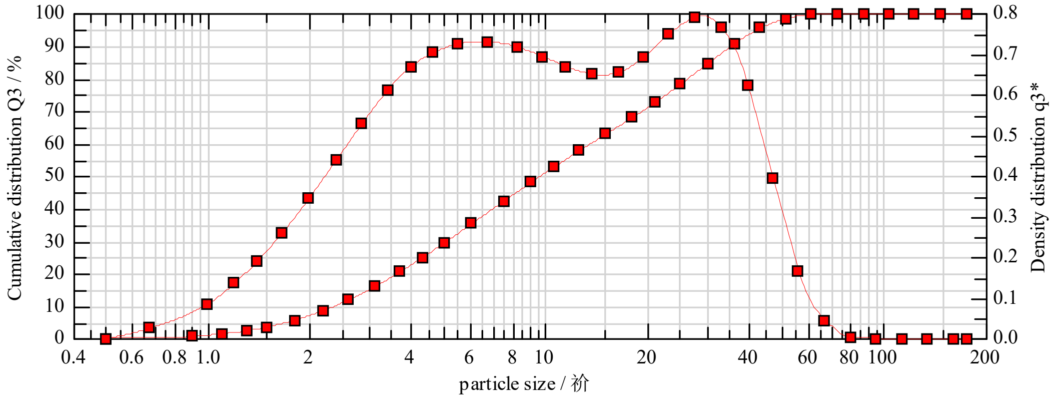
Cumulative distribution Q3 / %
Density distribution q3*
particle size / 祄
0 10 20 30 40 50 60 70 80 90 100
0.0 0.1 0.2 0.3 0.4 0.5 0.6 0.7 0.8
0.4 0.6 0.8 1.0 2 4 6 8 10 20 40 60 80 100 200

(c)

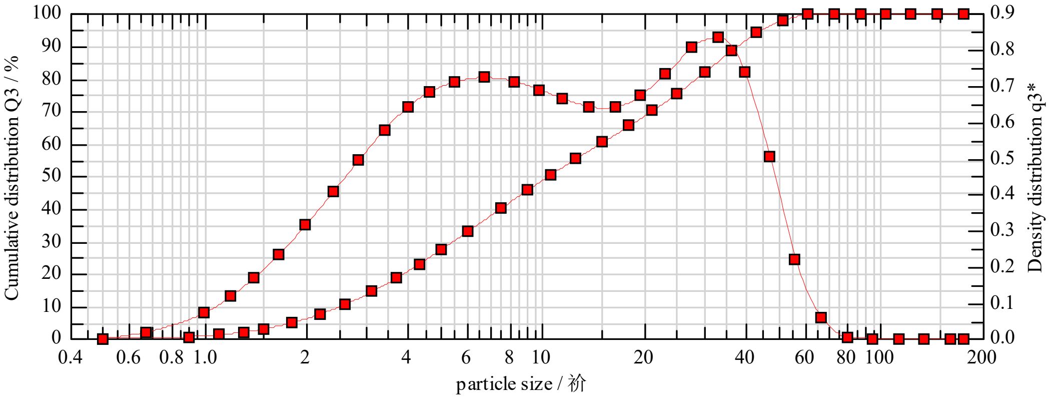

(d)

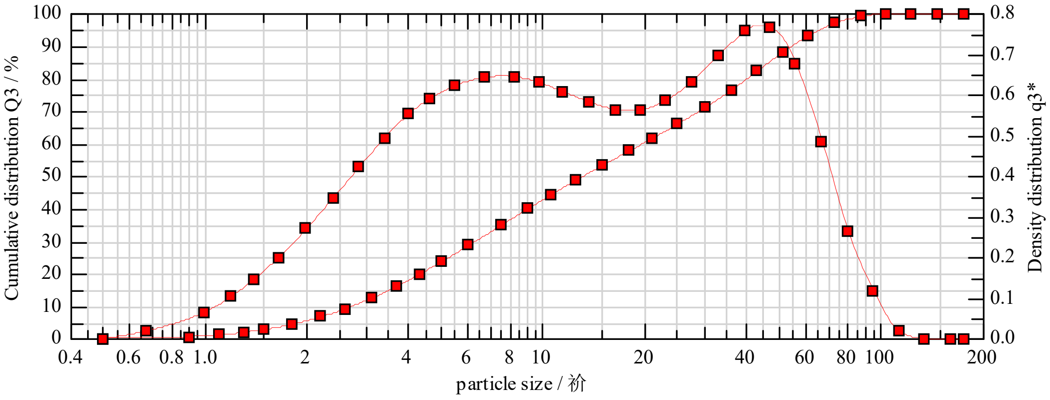

(e)

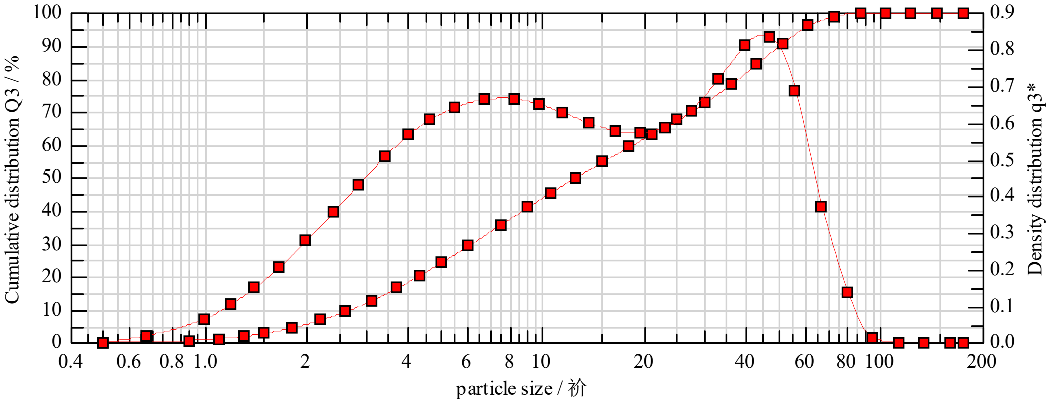

(f)

Figure S1. The examples of PSD graphs for **a.** LS, **b.** HS, **c.** LM, **d.** HM, **e.** LL, **f.** HL

2. Composition of Milk Chocolate Samples

The composition of milk chocolate samples is given in Table S2.

Table S2. Composition of milk chocolate samples (based on 100 grams)

Sample Code	Cocoa Solid	Saturated Fat	Total Fat	Sugar	PGPR	AMP
LS	30	16	30	55	0.05%	0.2%
LM	30	16	30	55	0.05%	0.2%
LL	30	16	30	55	0.05%	0.2%
HS	30	16	30	55	0.05%	0.8%
HM	30	16	30	55	0.05%	0.8%
HL	30	16	30	55	0.05%	0.8%
